# Coding Prony’s method in MATLAB and applying it to biomedical signal filtering

**DOI:** 10.1186/s12859-018-2473-y

**Published:** 2018-11-26

**Authors:** A. Fernández Rodríguez, L. de Santiago Rodrigo, E. López Guillén, J. M. Rodríguez Ascariz, J. M. Miguel Jiménez, Luciano Boquete

**Affiliations:** Grupo de Ingeniería Biomédica, Departamento de Electrónica, Universidad de Alcalá, Plaza de S. Diego, s/n, 28801 Alcalá de Henares, Spain

**Keywords:** Prony’s method, Matrix pencil, Least squares, Total least squares, Multifocal evoked visual potentials, Multiple sclerosis

## Abstract

**Background:**

The response of many biomedical systems can be modelled using a linear combination of damped exponential functions. The approximation parameters, based on equally spaced samples, can be obtained using Prony’s method and its variants (e.g. the matrix pencil method). This paper provides a tutorial on the main polynomial Prony and matrix pencil methods and their implementation in MATLAB and analyses how they perform with synthetic and multifocal visual-evoked potential (mfVEP) signals.

This paper briefly describes the theoretical basis of four polynomial Prony approximation methods: classic, least squares (LS), total least squares (TLS) and matrix pencil method (MPM). In each of these cases, implementation uses general MATLAB functions. The features of the various options are tested by approximating a set of synthetic mathematical functions and evaluating filtering performance in the Prony domain when applied to mfVEP signals to improve diagnosis of patients with multiple sclerosis (MS).

**Results:**

The code implemented does not achieve 100%-correct signal approximation and, of the methods tested, LS and MPM perform best. When filtering mfVEP records in the Prony domain, the value of the area under the receiver-operating-characteristic (ROC) curve is 0.7055 compared with 0.6538 obtained with the usual filtering method used for this type of signal (discrete Fourier transform low-pass filter with a cut-off frequency of 35 Hz).

**Conclusions:**

This paper reviews Prony’s method in relation to signal filtering and approximation, provides the MATLAB code needed to implement the classic, LS, TLS and MPM methods, and tests their performance in biomedical signal filtering and function approximation. It emphasizes the importance of improving the computational methods used to implement the various methods described above.

## Background

### Prony’s method

In 1795, Gaspard de Prony [1] proposed a method to explain the expansion of gases as a linear sum of damped complex exponentials of signals that are uniformly sampled. Prony’s method approximates a sequence of *N* = 2p equally spaced samples to a linear combination of p complex exponential functions with differing amplitudes, damping factors, frequencies and phase angles. The main contribution of this classic method is that it converts a non-linear approximation of exponential sums by solving a set of linear equations and a root-finding problem.

The conventional or polynomial Prony method consists of setting out an autoregressive model of order p that assumes that the value of sampled data x[n] depends linearly on the preceding *p* values in x. Solving this linear system of equations obtains the coefficients of the characteristic or Prony polynomial *φ*(*z*). The roots of this polynomial yield two of the parameters of the solution (damping factors and frequency) and provide a second system of equations to calculate the amplitude and phase of the p functions.

Prony’s original method exactly matched the curve of p exponential terms to a dataset of *N* = 2p elements. When *N* > 2p, the linear systems of equations are overdetermined and can be approximated by the least squares (LS) method [2]. The conventional least-squares method considers that in the linear system (**A.x** ≈ **b**), only **b** (observation vector) is contaminated by noise, while **A** (coefficient matrix) is noise-free. However, generally both matrix **A** and vector **b** are noise-perturbed (in Prony’s method, **A** and **b** share the same data source, see below) and, in this case, the total least-squares technique (TLS) [3] can be more advantageous.

In some cases, a problem with the Prony polynomial method is that it can be numerically unstable because of the steps that comprise the algorithm: solving an ill-conditioned matrix equation and finding the roots of a polynomial. When the number of exponentials is relatively high, the sensitivity of roots of the characteristic polynomial to perturbations of their coefficient is likewise high [4] and Prony’s method may be unstable.

Another alternative is to use the **matrix pencil method** (MPM). Although similar to Prony’s method, it consists of solving an eigenvalue problem rather than following the conventional two-step Prony method. It has been found through perturbation analysis and simulation that for signals with unknown damping factors the MPM is less sensitive to noise than the polynomial method [5].

In recent years, and due to advances in computing systems, Prony’s method has been successfully applied in various engineering sectors, such as electric power quality analysis [6], materials science [7], antennae [8], etc. In the biomedical field, the classic Prony method is used in [9] to process multifocal visual-evoked potentials (mfVEPs) to diagnose the early stages of multiple sclerosis (MS). The LS Prony method is used in [10] to estimate the parameters of the single event-related potential; the TLS is used in [11] to discriminate between three cardiac problems, and the MPM is used in [12–14].

Various programming languages are widely used in the scientific field. These languages include Python, a free and open-source high-level programming language [15, 16], and MATLAB®, a proprietary product.

MATLAB® is user-friendly and needs practically no formal programming knowledge [17]. As it implements a wide number and variety of functions (statistics, neural networks, graphics, wavelets, etc.), it is widely accepted as a development platform for numerical software by a significant portion of the computational science and engineering community [18–20]. Its open availability ensures reproducibility and knowledge exchange.

## Objectives

This paper presents a tutorial on implementation in MATLAB of two families of Prony methods: the **polynomial method** (classic and extended — LS and TLS) and the **matrix pencil method**. It presents an overview of the mathematical bases of each method and implements them in MATLAB using the functions directly available. The results produced by the different methods when approximating synthetic signals are obtained and filtering of mfVEP records is implemented in the Prony domain. The Discussion section provides information on possible ways of mitigating the ill-conditioning problems associated with several of the resolution phases of the Prony methods.

## Implementation

### Polynomial method

A data sequence *x*[*n*] (*n* = 1,…N) can be represented by the sum of *p* complex parameters (order p) according to the following expression:

1 $$ x\left[n\right]=\sum \limits_{k=1}^p{A}_k{e}^{j{\theta}_k}\cdotp {e}^{\left({\alpha}_k+j2\pi {f}_k\right){T}_s\left(n-1\right)}=\sum \limits_{k=1}^p{h}_k\cdotp {z}_k^{\left(n-1\right)} $$

Approximation of signal *x*[*n*] occurs in *p* components, in which *A*_*k*_ is the initial amplitude in the same units as *x*[*n*], *α*_*k*_ is the damping factor in seconds^−1^, *f*_*k*_ is the frequency in Hertz, T_S_ is the sampling period (in seconds) of signal *x*[*n*] and *θ*_*k*_ is the initial phase in radians. Therefore, signal *x*[*n*] is characterized by the parameters *A*_*k*_, *α*_*k*_, *f*_*k*_ and *θ*_*k*_ (k = 1,…,p). *h*_*k*_ is the time-independent component and *z*_*k*_ is an exponential and time-dependent component (poles).

Equation 1 is the expression of the general solution of a homogeneous linear difference equation, if the *p* roots are different [21]. In order to find that equation we have to construct its characteristic equation, which is

2 $$ \varphi (z)=\prod \limits_{k=1}^p\left(z-{z}_k\right)=\sum \limits_{k=0}^pa\left[k\right]{z}^{p-k};\kern3.75em a\left[0\right]=1 $$

where the characteristic roots are the parameters *z*_*k*_ in Eq. 1.

Demonstration of the Prony approximation method is found in [22]. Practical implementation requires performance of the following steps:

**Step 1:** Solve the linear prediction model constructed by the observed dataset and the obtained coefficients (a [1]…a[p]) of the characteristic polynomial. An autoregressive model of order p considers that the value of x[n] depends linearly on the preceding *p* values in x. Equation 1 can be rewritten as a linear prediction model according to the matrix system **T**_pxp_.**a**_px1_ = − **x**_px1_:

3 $$ \left(\begin{array}{cccc}x\left[p\right]& x\left[p-1\right]& \cdots & x\left[1\right]\\ {}x\left[p+1\right]& x\left[p\right]& \cdots & x\left[2\right]\\ {}\vdots & \vdots & \ddots & \vdots \\ {}x\left[2p-1\right]& x\left[2p-2\right]& \cdots & x\left[p\right]\end{array}\right)\left(\begin{array}{c}a\left[1\right]\\ {}a\left[2\right]\\ {}\vdots \\ {}a\left[p\right]\end{array}\right)=-\left(\begin{array}{c}x\left[p+1\right]\\ {}x\left[p+2\right]\\ {}\vdots \\ {}x\left[2p\right]\end{array}\right) $$

Where.

**a**: Linear prediction coefficients vector.

**x**: Observation vector.

**T**: Forward linear prediction matrix (a square Toeplitz matrix).

Solving this linear system (3) reveals that the values of **a** are the coefficients of the characteristic or Prony polynomial *φ*(*z*).

**Step 2:** Find the roots of the characteristic or Prony polynomial formed from the linear prediction coefficients.

Solving an equation in finite differences is achieved by finding the roots of the characteristic polynomial. As vector **a** is known from (3), the roots z_k_ of the polynomial *φ*(*z*) can be computed to obtain the damping factor (*α*_*k*_) and frequency (*f*_*k*_).

4 $$ {\alpha}_k=\frac{\ln \left|{z}_k\right|}{T_s} $$

5 $$ {f}_k=\frac{\tan^{-1}\left[\frac{\mathit{\operatorname{Im}}\left({z}_k\right)}{\mathit{\operatorname{Re}}\left({z}_k\right)}\right]}{2\pi {T}_s} $$

**Step 3**: Solve the original set of linear equations to yield the estimates of the exponential amplitude and sinusoidal phase.

First, the initial system of equations (**Z**_pxp_.**h**_px1_ = **x**_px1_) is solved:

6 $$ \left(\begin{array}{cccc}{z}_1^0& {z}_2^0& \cdots & {z}_p^0\\ {}{z}_1^1& {z}_2^1& \cdots & {z}_p^1\\ {}\vdots & \vdots & \ddots & \vdots \\ {}{z}_1^{p-1}& {z}_2^{p-1}& \cdots & {z}_p^{p-1}\end{array}\right)\left(\begin{array}{c}{h}_1\\ {}{h}_2\\ {}\vdots \\ {}{h}_P\end{array}\right)=\left(\begin{array}{c}x\left[1\right]\\ {}x\left[2\right]\\ {}\vdots \\ {}x\left[p\right]\end{array}\right) $$

The **h**_**k**_ values yield the amplitude (*A*_*k*_) and phase (*θ*_*k*_):

7 $$ {A}_k=\left|{h}_k\right| $$

8 $$ {\theta}_k={\tan}^{-1}\left[\frac{\mathit{\operatorname{Im}}\left({h}_k\right)}{\mathit{\operatorname{Re}}\left({h}_k\right)}\right] $$

The classic Prony method (N = 2p) obtains an exact fit between the sampled data points and the exponentials if matrices **T** and **Z** are non-singular. However, in many practical cases N > 2p and, in this situation, both systems are overdetermined (more equations than unknowns) and can be approximated using the LS or TLS methods.

#### Least squares

In general, given the overdetermined linear system: **A x** ≈ **b** with **A** ∈ *ℂ*^mxn^, **b** ∈ *ℂ*^mx1^, **x** ∈ *ℂ*^nx1^, m > n; being **A** the data matrix and **b** the observation vector, the least squares solution **x**_LS_ is given by the normal equation:

9 $$ {\mathbf{x}}_{\mathrm{LS}}={\left({\mathbf{A}}^{\mathrm{H}}\mathbf{A}\right)}^{-1}{\mathbf{A}}^{\mathrm{H}}\ \mathbf{b}={\mathbf{A}}^{+}\ \mathbf{b} $$

H represents the Hermitian conjugate of a matrix and **A**^**+**^ is the Moore–Penrose pseudoinverse matrix of **A**. In practice, the normal equation is rarely used, as methods based on QR decomposition or singular value decomposition (SVD), among others, are preferable.

#### Total least squares

Solution of the system **A x** ≈ **b** by the total least-squares method is a generalization of the LS approximation method when the data matrix **A** and observation vector **b** are contaminated with noise. In Prony’s method, eqs. 3 and 6 are constructed from the measured signals. The basic total least-squares algorithm is [3]:

**C** ≔ [**A** : **b**], matrix **A** augmented (expansion by columns) by vector **b** (**C** ∈ *ℂ*^mx(n + 1)^). SVD of **C** matrix is then performed:

10 $$ \mathbf{C}=\mathbf{U}\boldsymbol{\Sigma } {\mathbf{V}}^{\mathbf{H}} $$

The matrices **U**_m × m_ (left singular vector matrix) and **V**_(n + 1) × (n + 1)_ (right singular vector matrix) are orthonormal (**U**^H^**U** = **UU**^H^ = **I**_*m*_,  **V**^H^**V** = **VV**^H^ = **I**_*n* + 1_) and **Σ**_m × (n + 1)_ = diag(σ_1_, σ_2_, …σ_min {m,   n + 1}_)) being *σ*_1_ ≥ *σ*_2_… ≥ *σ*_min {*m*,   *n* + 1}_ the singular values of **C**.

The structure of **V** is as follows:

11 $$ \mathbf{V}=\left[\begin{array}{ccc}{\mathrm{v}}_{1.1}& \cdots & {\mathrm{v}}_{1,\left(\mathrm{n}+1\right)}\\ {}\vdots & \ddots & \vdots \\ {}{\mathrm{v}}_{\left(\mathrm{n}+1\right),1}& \cdots & {\mathrm{v}}_{\left(\mathrm{n}+1\right),\left(\mathrm{n}+1\right)}\end{array}\right] $$

The TLS solution exists if *v*_(*n* + 1), ( *n* + 1)_ ≠ 0 [23] and, moreover it is unique if *σ*_*n*_ ≠ *σ*_*n* + 1_:

12 $$ {\mathbf{x}}_{\mathrm{T}\mathrm{LS}}=\kern0.5em -\frac{1}{{\mathrm{v}}_{\left(\mathrm{n}+1\right),\left(\mathrm{n}+1\right)}}\kern0.5em {\left[{\mathrm{v}}_{1,\left(\mathrm{n}+1\right)},{v}_{2,\left(n+1\right)}\kern0.5em \cdots \kern0.5em {\mathrm{v}}_{\mathrm{n},\left(\mathrm{n}+1\right)}\right]}^{\mathrm{T}} $$

Algorithms in which the solution does not exist or is not unique are considered in detail in [24].

#### Implementation in MATLAB of the polynomial method

The code presented was developed and tested under MATLAB R2016b. **Code 1** presents implementation in MATLAB of a function to perform the Prony approximation using the three polynomial methods mentioned above. The function is defined as follows:

**function [Amp,alpha,freq,theta] = polynomial_method (x,p,Ts,method)**

The sampled data are given in vector x; *p* is the number of terms to obtain in the approximation, Ts is the sampling time of the signal and classic, LS or TLS indicates the method used to solve the problem. The function returns the parameters (Amp, alpha, freq, theta) resulting from the approximation.

First, the sample length is obtained (**N = length(x)**) and consistency between the parameter method, *p* and the sample data length is checked.

**Step 1.**

Coding the linear system of Eq. 2 takes into account that the MATLAB function ***T*** *= toeplitz(c,r)* creates non-symmetrical Toeplitz matrix **T** (dimensions *p* × *p* under the classic method and (*N* − *p*) × *p* under the overdetermined methods), having *c* as its first column and *r* as its first row, achieved by the following instruction:

**T = toeplitz (x(p:N-1), x(p:-1:1)**);

The solution of this system of eqs. (**T**.**a** = −**x**) for the classic and LS methods is obtained in MATLAB using the matrix left division (also known as backslash) operator. If **T** is square and if it is invertible, the backslash operator solves the linear equations using the QR method. With an overdetermined system, LS should be used. The backslash operator is a collection of algorithms used to solve a linear system [25], selected according to the characteristics of matrix **T**. Taking into account that vector **x** is a matrix column:

**a = − T \ x(p + 1:N);**

In the case of the TLS option, the function **a = tls(T,-x(p + 1:N));** is called (**Code 2**).

**Step 2.**

The p roots of the polynomial are now obtained:

$$ {z}^p+a\left[1\right]{z}^{p-1}+\dots +a\left[p\right]=0 $$

The MATLAB instruction *r = roots(c)* returns a column vector whose elements are the roots of the polynomial c. Row vector c contains the coefficients of a polynomial, ordered in descending powers. If c has n + 1 components, the polynomial it represents is c_1_s^n^ + … + c_n_s + c_n + 1_.

The input vector for the **roots** function must be a row vector and must contain the element *a*[0] = 1, which was not obtained in the previous solution. Its implementation is therefore.

**c = transpose([1; a]);**

**r = roots(c);**

Based on r, and having defined the acquisition period Ts, it is possible to find the values of the damping factor (*α*_*k*_) and frequency (*f*_*k*_):

**alpha = log(abs(r))/Ts;**

freq = atan2(imag(r),real(r))/(2*pi*Ts);

log is the Napierian logarithm and atan2 returns the four-quadrant inverse tangent.

**Step 3: Obtain complex parameters** ***h***_***k***_ **from roots** ***z***_***k***_ **.**

The number of equations (**len_vandermonde**) employed for the solution is set (p in classic and N in overdetermined systems) and the data matrix for the system of equations is constructed (6):

**Z = zeros(len_vandermonde,p);**

**for** ***i*** **= 1:length(r).**

**Z(:,i) = transpose(r(i).^(0:len_vandermonde-1));**

**End**

Finally, the following is solved:

**h = Z \ x(1:len_vandermonde);**

In the case of the TLS option, the function **h = tls(Z, x(1**: **len_vandermonde));** (**Code 2**) is called. In the TLS algorithm, SVD is used. The infinite values therefore have to be converted into maximum representative values beforehand otherwise the SVD function will yield an error.

The solutions yield the initial amplitude (*A*_*k*_) and initial phase (*θ*_*k*_) values:

**Amp = abs(h);**

**theta = atan2(imag(h),real(h));**

The function that solves a linear system using the TLS method (**Code 2**) receives as arguments matrices **A** and **b**, which define the linear system to solve: **Function x = tls(A,b).** The number of columns in matrix **A** is obtained (**[~,n] = size(A);**) and augmented matrix **C** (**C = [A b]**) is constructed while matrix **V** of the SVD decomposition is obtained via the instruction **[~,~,V] = svd(C)**; the TLS solution (if it exists) is obtained by applying the formula (12) to matrix **V**.

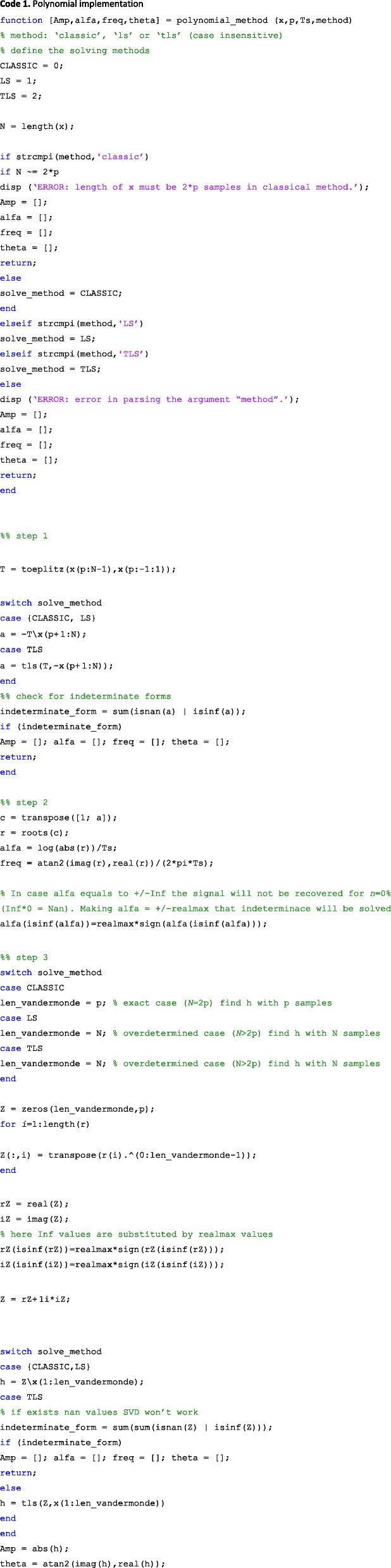

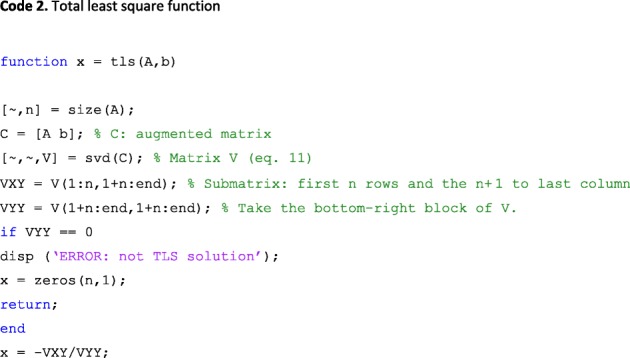





### Matrix pencil method

Steps 1 and 2 of the polynomial method yield the roots of the characteristic polynomial that coincide with the signal poles z_k_. An alternative solution is to use the MPM to find *z*_*k*_ directly by solving a generalized eigenvalue problem.

In general, given two matrices **Y**_**1**_ ∈ *ℂ*^mxn^,  **Y**_**2**_ ∈ *ℂ*^mxn^, the set of matrices of the form ***Y***_**2**_ − *λ****Y***_**1**_ (**λ** ∈ *ℂ*) is a matrix pencil [26].

In our case, to implement MPM a rectangular Hankel matrix **Y** is formed from the signal (*x*[*n*], *n* = 1,…N), where, in this method, *p* is the pencil parameter:

13 $$ \mathbf{Y}={\left(\begin{array}{ccccc}\mathrm{x}\left[1\right]& \mathrm{x}\left[2\right]& \cdots & \mathrm{x}\left[\mathrm{p}\right]& \mathrm{x}\left[\mathrm{p}+1\right]\\ {}\mathrm{x}\left[2\right]& \mathrm{x}\left[3\right]& \cdots & \mathrm{x}\left[\mathrm{p}+1\right]& \mathrm{x}\left[\mathrm{p}+2\right]\\ {}\vdots & \vdots & \ddots & \vdots & \vdots \\ {}\mathrm{x}\left[\mathrm{N}-\mathrm{p}\right]& \mathrm{x}\left[\mathrm{N}-\mathrm{p}+1\right]& \cdots & \mathrm{x}\left[\mathrm{N}-1\right]& \mathrm{x}\left[\mathrm{N}\right]\end{array}\right)}_{\left(\mathrm{N}-\mathrm{p}\right)\times \left(\mathrm{p}+1\right)} $$

This matrix is used to create matrices **Y**_1_ and **Y**_2_. **Y**_1_ is constructed by eliminating the last column of **Y** while **Y**_2_ is constructed by eliminating the first column of **Y**:

14 $$ {\mathbf{Y}}_1={\left(\begin{array}{cccc}\mathrm{x}\left[1\right]& \mathrm{x}\left[2\right]& \cdots & \mathrm{x}\left[\mathrm{p}\right]\\ {}\mathrm{x}\left[2\right]& \mathrm{x}\left[3\right]& \cdots & \mathrm{x}\left[\mathrm{p}+1\right]\\ {}\vdots & \vdots & \ddots & \vdots \\ {}\mathrm{x}\left[\mathrm{N}-\mathrm{p}\right]& \mathrm{x}\left[\mathrm{N}-\mathrm{p}+1\right]& \cdots & \mathrm{x}\left[\mathrm{N}-1\right]\end{array}\right)}_{\left(\mathrm{N}-\mathrm{p}\right)\times \mathrm{p}} $$

15 $$ {\mathbf{Y}}_2={\left(\begin{array}{cccc}\mathrm{x}\left[2\right]& \cdots & \mathrm{x}\left[\mathrm{p}\right]& \mathrm{x}\left[\mathrm{p}+1\right]\\ {}\mathrm{x}\left[3\right]& \cdots & \mathrm{x}\left[\mathrm{p}+1\right]& \mathrm{x}\left[\mathrm{p}+2\right]\\ {}\vdots & \ddots & \vdots & \vdots \\ {}\mathrm{x}\left[\mathrm{N}-\mathrm{p}+1\right]& \cdots & \mathrm{x}\left[\mathrm{N}-1\right]& \mathrm{x}\left[\mathrm{N}\right]\end{array}\right)}_{\left(\mathrm{N}-\mathrm{p}\right)\times \mathrm{p}} $$

Where M is the real number of poles of function x[n], if M ≤ p ≤ (N − M) is fulfilled, the poles z_k_ (k = 1,….p) are the generalized eigenvalues of the matrix pencil **Y**_2_ − λ**Y**_1_ [27]; matrices **Y**_1_ and **Y**_2_ are ill-conditioned and therefore the QZ-algorithm is not stable enough to yield the generalized eigenvalues [5]. It is more efficient to obtain the values of z_k_ from the following expression:

16 $$ {z}_k=\mathrm{eigenvalues}\ \left({\mathbf{Y}}_1^{+}{\mathbf{Y}}_2\right) $$

Where ${\mathbf{Y}}_1^{+}$ is the Moore–Penrose pseudoinverse matrix of **Y**_1_, defined as:

17 $$ {\mathbf{Y}}_1^{+}={\left[{\mathbf{Y}}_1^{\mathrm{H}}{\mathbf{Y}}_1\right]}^{-1}{\mathbf{Y}}_1^{\mathrm{H}} $$

The values *z*_*k*_ yield the parameters *α*_*k*_ and frequency *f*_*k*_ (Equations 5 and 6); The final step coincides with Step 3 of the Prony polynomial method: solving the system **Z**_pxp_.**h**_px1_ = **x**_px1_ and obtaining *A*_*k*_ and *θ*_*k*_ (Equations 8 and 9).

Coding of the MPM in MATLAB is done in **Code 3**, the function call being.

**Function [Amp,alpha,freq,theta] = matrix_pencil (x,p,Ts)**

The first step is to obtain the matrix ***Y*** then, based on that, matrices ***Y***_**1**_ and ***Y***_**2**_. To achieve this, the following instruction is employed:

**Y = hankel (x(1:end-p), x(end-p:end)**);

To obtain ***Y***_**1**_, the last column is eliminated.

**Y1 = Y (:,1:end-1);**

To obtain ***Y***_**2**_, the first column is eliminated.

**Y2 = Y (:,2:end);**

The eigenvalues are obtained (Equation 16).

l = eig (pinv(Y1)*Y2);

**eig (A)** is a function that returns the eigenvalues of **A** while **pinv(A)** yields the Moore–Penrose pseudoinverse matrix of **A** which, in this case, corresponds to the expression in Equation 17.

The frequency (*f*_*k*_) and damping factor (*α*_*k*_) values are obtained from the eigenvalues in the same way as the roots are obtained in the polynomial method:

**alpha = log(abs(l))/Ts;**

freq = atan2(imag(l),real(l))/(2*pi*Ts);

To calculate the initial amplitude and phase values (*A*_*k*_ and *θ*_*k*_), the steps followed are exactly the same as in the polynomial method.

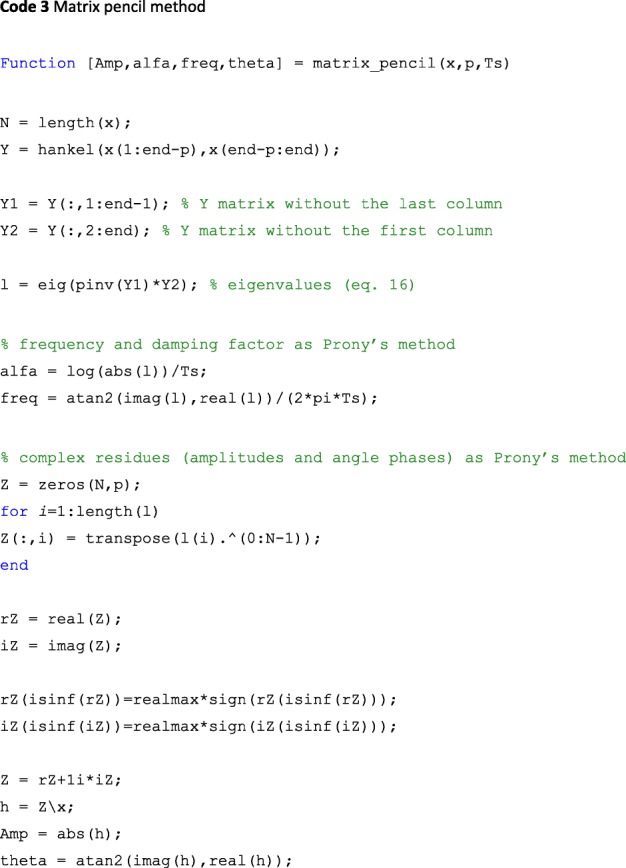



## Results

The methods described are applied in two situations: a) approximation of synthetic signals and b) filtering of mfVEP signals.

### Synthetic functions

1 000 Functions are generated (*g*_*i*_[*n*]) with *N* = 1 024 points each (*i* = 1, …1 000; *n* = 0, …1 023), according to the following expression

18 $$ {g}_i\left[n\right]=\sum \limits_{k=0}^9{A}_k.{e}^{\alpha_k.n.{T}_S}.\cos \left(2.\pi .{f}_k.n.{T}_S+{\theta}_k\right) $$

The parameters of the functions have a uniform random distribution at the following intervals: *A*_*k*_ ∈ [1, 10];   *α*_*k*_ ∈ [0, ***−***4], *f*_*k*_ ∈ [1, 31],  *f*_*i*_ ≠ *f*_*j*_;   *θ*_*k*_ ∈ [−*π*, *π*] and *f*_0_ = 0.

Due to the possible existence of numerical errors in the computational approximation of the functions it is advisable to evaluate the error between the original function (*g*_*i*_[*n*]) and its approximation ($\overset{\sim }{g_i}$) using Prony’s method. The precision of the approximation obtained from the normalized root-mean-square error is used:

19 $$ G=1-\frac{\parallel {g}_i\left[n\right]-\overset{\sim }{g_i\left[n\right]}\parallel }{\parallel {g}_i\left[n\right]-\overline{\kern0.75em {g}_{i\kern0.5em }}\parallel } $$

‖.‖ indicates the 2-norm and $\overline{\ {g}_i}$ is the mean of the reference signal.

If for a certain function *G* ≥ 0.60 is fulfilled, the approximation is considered correct. Table 1 shows the number of functions correctly approximated by the Prony LS, Prony TLS and MPM methods and for the two different parameters (N, p).

**Table 1:** Result of approximation of synthetic functions

Number of functions *g*_*i*_[*n*] correctly approximated				
N	p	LS	TLS	MPM
1024	30	902	811	990
	40	868	499	1000
	50	826	499	1000
	100	997	322	1000
	150	1000	315	1000
	200	1000	375	1000
	250	1000	358	1000
	300	1000	288	1000
	400	1000	224	1000
	500	999	137	1000
512	30	941	741	1000
	40	974	660	1000
	50	996	682	1000
	60	999	618	1000
	70	1000	544	1000
	100	1000	565	1000
	150	1000	622	1000
	200	1000	579	1000
	220	1000	517	1000
	250	999	516	1000
256	30	984	909	1000
	40	998	872	1000
	50	998	855	1000
	60	1000	826	1000
	70	1000	778	1000
	80	1000	862	1000
	90	1000	827	1000
	100	1000	758	1000
	110	1000	733	1000
	120	996	758	1000
128	20	994	995	994
	30	1000	960	1000
	40	1000	956	1000
	50	1000	931	1000
	60	1000	910	1000
64	20	1000	1000	999
	25	1000	969	1000
	30	1000	970	1000
Average value per method		$\overline{\mathrm{LS}}=986.08$	$\overline{\mathrm{TLS}}=677.39$	$\overline{\mathrm{MPM}}=999.55$

None of the methods implemented works 100% correctly (*G* ≥ 0.60 for the 1000 *g*_*i*_[*n*] functions in all the situations tested). If the mean number of functions well-approximated by each method is considered, the best result is obtained with MPM ($\overline{MPM}=999.55$) and the worst is obtained with TLS ($\overline{TLS}=677.39$). The LS method yields the correct approximation in 60.52% of cases, the TLS method in 2.63% of cases and the MPM method in 92.10% of cases tested in this experiment.

In general, the results obtained using LS and MPM are very similar, as the MATLAB roots(·) function generates the companion matrix of the polynomial and uses the QR-algorithm to obtain its eigenvalues.

**Fig. 1:**
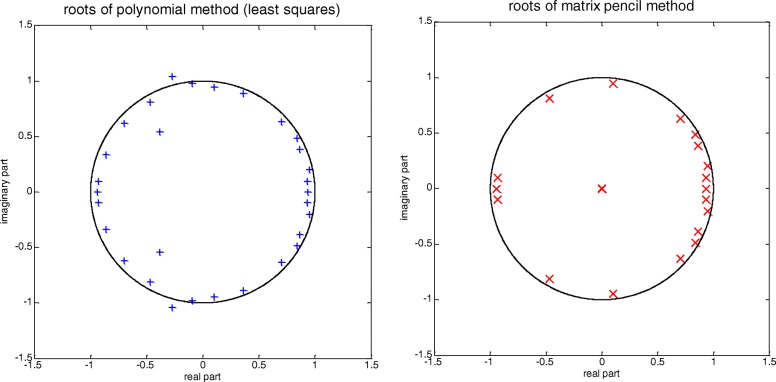
Poles obtained using the polynomial (LS) and MPM methods

Figure 1 shows the roots obtained using the LS and MPM methods for one of the *g*_*i*_[*n*] signals (*N* = 256, *p* = 30). The correct number of roots for signal *g*_*i*_[*n*] is *M* = 19; in both examples, *p* = 30 roots are obtained, though with the MPM method 12 roots are equal to 0. This is because in the LS method the range of the companion matrix is always equal to *p* and, consequently, *p* roots are obtained. In the MPM method, the range of matrix $\left({\mathbf{Y}}_1^{+}{\mathbf{Y}}_2\right)$ is less than or equal to p ($\mathrm{r}=\operatorname{rank}\left({\mathbf{Y}}_1^{+}{\mathbf{Y}}_2\right)\le p$) and r roots other than zero and (p-r) roots equal to 0 are obtained [5]. In the example shown, *r* = 18 is fulfilled. The differences in the results between the two methods are evident in Step 3 and are due to computational errors.

### mfVEP filtering

The mfVEP technique [28] can be used to obtain the electrophysiological response of the primary visual cortex to stimuli produced in a large number (e.g. 60) of sectors of the visual field. Generation of the visual stimulus is governed by the same pseudorandom sequence [29] used to separate the individual responses of each sector from the continual EEG recording obtained using electrodes. Analysis of mfVEP signals is employed in diagnosis and study of patients with glaucoma, amblyopia, nerve optic drusses, optic neuritis, multiple sclerosis and other pathologies.

The aim of this test is to evaluate whether mfVEP signal filtering in the Prony domain improves the separation between the signals of control subjects and the signals of patients with MS. It uses the signal-to-noise ratio (SNR) of the records as the parameter. The discrimination factor is evaluated using the area under the ROC curve (AUC). The results achieved by applying the conventional method to mfVEP records are then compared: signals filtered using the discrete Fourier transform (DFT) between 0 and 35 Hz and the signals filtered in the Prony domain.

This experiment uses a database of mfVEP signals captured from 28 patients (age 34.39 ± 10.09 years, 7 males and 21 females) diagnosed with MS according to the McDonald criteria; the signals were obtained from 44 eyes in 22 control subjects (age 30.20 ± 7.55 years, 10 males and 12 females) with normal ophthalmologic and neurological examination results. The study protocol adhered to the tenets of the Declaration of Helsinki and was approved by the local Institutional Review Board (Comité de Ética en Investigación Clínica del Hospital Universitario Príncipe de Asturias, Alcalá de Henares, Spain). Written informed consent was obtained from all participants.

mfVEP signals were recorded monocularly with VERIS software 5.9 (Electro-Diagnostic Imaging, Inc., Redwood City, CA). The visual stimulus was a scaled dartboard with a diameter of 44.5 degrees, containing 60 sectors, each with 16 alternating checks. The luminance for the white and black checks were 200 and < 3 cd/m^2^, respectively. The checks in each sector were reversed in contrast using a pseudorandom sequence (m-sequence) at a frame rate of 75 Hz.

The mfVEP signals were obtained using gold cup electrodes (impedance < 2 KΩ). The reference electrode was positioned on the inion (E_R_) and the ground electrode on the forehead. The active electrodes were placed 4 cm above the inion (E_A_) and 1 cm above and 4 cm either side of the inion (E_B_, E_C_). The difference between the signals of the active electrodes was used to obtain channels CH_1_ = E_A_-E_R_, CH_2_ = E_B_-E_R_ and CH_3_ = E_C_-E_R_. Three additional derived channels were obtained (CH_4_ = CH_1_-CH_2_, CH_5_ = CH_1_-CH_3_, CH_6_ = CH_2_-CH_3_). Therefore, the data from 6 channels were processed. In the analogue phase, the signals were amplified at a gain of 10^5^ at a bandwidth between 3 and 100 Hz. The sampling frequency was 1200 Hz, obtaining 600 samples in each recording (length 500 ms).

The conventional signal-processing method consists of bandpass filtering between 0 and 35 Hz using the fast Fourier transform; these signals are denominated X_DFT_.

One method for determining the intensity of the mfVEP records is to use the signal-to-noise ratio, defined by the following expression:

20 $$ \mathrm{SNR}=\frac{{\mathrm{RMS}}_{45-150\ \mathrm{ms}}}{\mathrm{mean}\ \left({\mathrm{RMS}}_{325-430\ \mathrm{ms}}\right)}; $$

In an mfVEP, the physiological response to the stimulus presents in the 45–150 ms interval (signal window) following onset. In the 325–430 ms interval (noise window) only noise is considered to be recorded. RMS_45–150 ms_ and RMS_325 − 430 ms_ are the root mean square (RMS) amplitudes in the signal window and noise window, respectively.

**Fig. 2:**
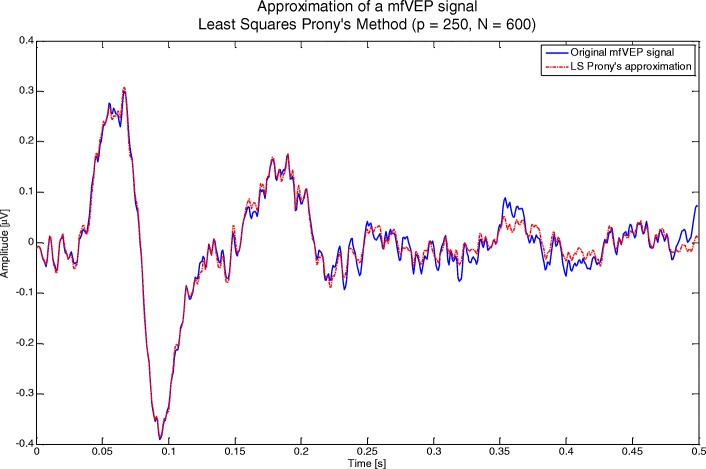
Example of approximation of an mfVEP signal using Prony’s method (LS)

**Fig. 3:**
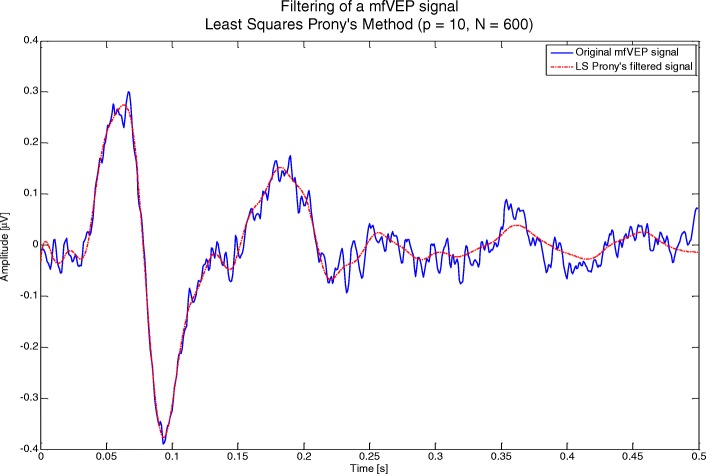
Example of Prony filtering of an mfVEP signal

Signal processing using Prony’s method is carried out in the following steps:

1. The Prony approximation is obtained (X_LS_, X_TLS_, X_MPM_, with *p* = 250, *N* = 600) for the X_DFT_ signals. The number of MS signals is N_MS_ = 20,160 (28 × 2 × 60 × 6) and the number of control signals is N_CONTROL_ = 15,840 (22 × 2 × 60 × 6).

2. Correct approximation of the X_DFT_ signal is checked against the expression shown in Equation 19 and considering G ≥ 0.45. Figure 2 shows an example of a signal approximated using the LS method.

3. The correctly approximated signals are bandpass-filtered in the Prony domain, selecting the 10 lowest-frequency components. The MATLAB code is shown in Code 4. Figure 3 shows an example of a filtered signal.

4. The SNR value of the X_DFT_ and Prony-filtered signals (X_LS_F_, X_TLS_F_, X_MPM_F_) is obtained and the discrimination value between the signals of subjects with MS and control subjects is calculated.

Similar to the case of the synthetic signals, the LS method only correctly approximated a low percentage of records (48.79% of the control records and 42.90% of the MS records) (Table 2). The LS and MPM methods yielded the same results, achieving correct approximation in over 99% of cases. The signal intensity value in the control subjects is greater than in the subjects with MS. Filtering the signals using the conventional method yields an AUC value of 0.6538; using the TLS method yields practically the same result (AUC = 0.6472) while using the LS and MPM methods yields a value of 0.7055. This example shows that filtering in the Prony domain can increase the capacity to discriminate between the signals of control subjects and those of patients with MS.

**Table 2:** Results of filtering mfVEP signals (N = 600, p = 250, *T*_*s*_ = 1/1200 *s*)

	Well-approximated control signals (%)	Well-approximated MS signals (%)	SNR_CONTROLS_	SNR_MS_	AUC
**DFT**	–	–	3.59 ± 2.89	2.44 ± 2.11	0.6538
**LS**	99.57%	99.91%	4.95 ± 4.19	2.85 ± 2.49	0.7055
**TLS**	48.79%	42.90%	3.65 ± 2.89	2.54 ± 2.24	0.6472
**MPM**	99.57%	99.91%	4.95 ± 4.19	2.85 ± 2.49	0.7055

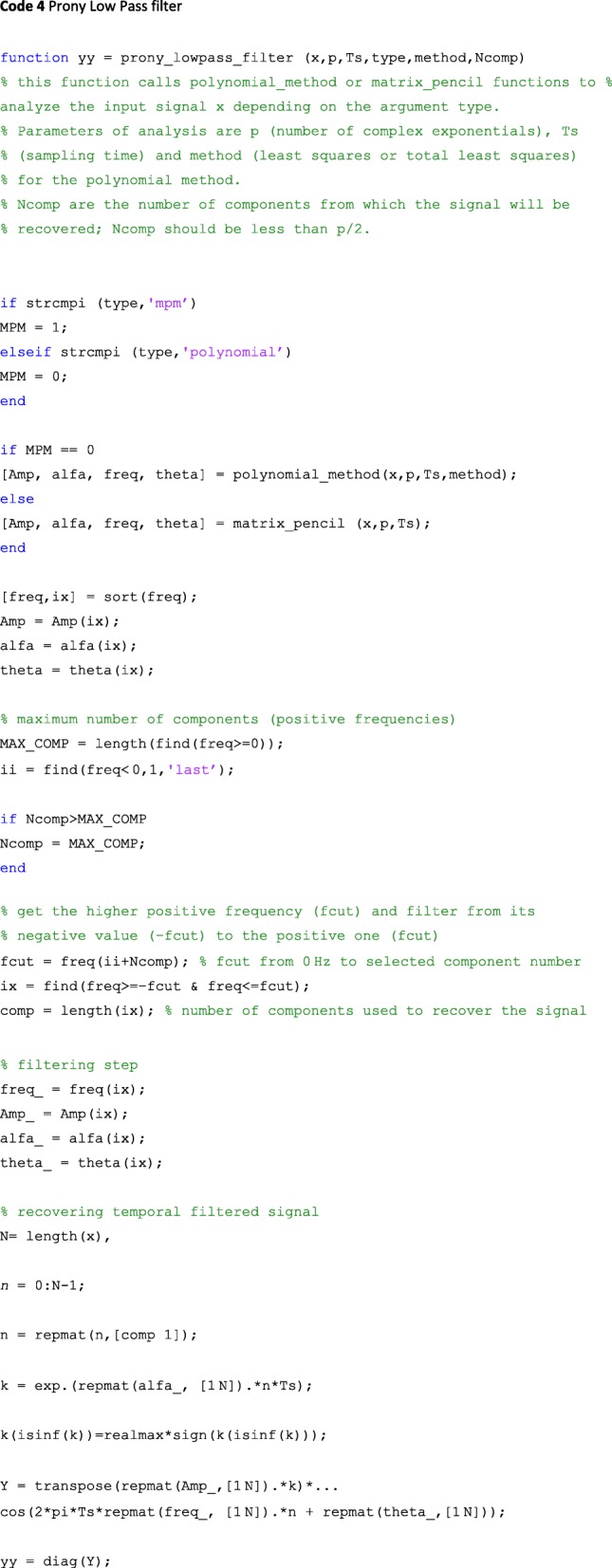



## Discussion

In this paper we have used general MATLAB functions to implement the principal methods of function approximation based on the linear combination of exponentials: the polynomial Prony method (classic, LS and TLS) and the matrix pencil method. In the polynomial method, signal poles (frequencies and damping factors) are found as roots of a polynomial while the MPM obtains the poles by finding the eigenvalues of a matrix pencil.

Currently, the most common method is Fourier analysis, which represents a signal as a summation of continuous undamped sinusoidal functions with frequency and integer times the fundamental frequency (harmonics). In contrast, the p components of a Prony series may be complex exponentials. In general, the Prony spectrum will be non-uniformly spaced in the frequency scale (as it is one of the estimated parameters), depending on the observed data [30].

Prony modelling produces higher frequency resolution than DFT methods due to its reliance on autoregressive modelling [31]. Another advantage is that it is a natural transformation for impulse responses since it uses damped sinusoids as a basis and therefore representation is efficient in terms of the number of coefficients required [32].

Not all mathematical signals can be approximated using Prony’s method [33] and computational finite arithmetic also generates errors. Consequently, the results of computational implementation of the Prony methods depend on the characteristics and number of points of the signal to be interpolated, on the p number of functions and on the use of computational procedures not susceptible to ill-conditioning issues. Furthermore, these potentially ill-conditioned operations are concatenated, thereby increasing the instability issues. For example, since the second step of Prony’s method is an ill-conditioned problem and round-off errors must exist for the linear prediction parameters to be computed in the first step, the estimation of z_k_ in the second step of Prony’s method can contain significant error [34].

In our experimental approximation of synthetic functions, the best result was obtained using the MPM and LS methods, while the effectiveness of the TLS method was shown to be highly dependent on the number of points and on the p number of functions (Table 1). In some cases, when the number of exponentials is relatively high, the sensitivity of roots of the characteristic polynomial to perturbations of their coefficient is likewise high [4] and Prony’s method may be unstable.

In a second experiment, we low-pass-filtered mfVEP signals in the Prony domain in order to evaluate the improvement in the capacity to discriminate between signals of control subjects and those of MS patients. Selecting the first 10 components of each record reveals that the AUC value between the signals of healthy subjects and those of MS subjects increases by between 0.3% and 4.7% depending on the method compared. The smallest improvement was obtained with the TLS method and the greatest improvement with the LS and MPM methods.

Coding in MATLAB used the functions directly available in this programming language. However, these evidently have their computational limitations and could be replaced with better alternatives. Various aspects that could improve the code presented in this paper are discussed below.

### Solution of linear systems

Solution of the linear systems using the classic and LS methods was implemented with the MATLAB *mldivide* (\) operator. Although the *mldivide* operator is valid for most cases (it selects between the LU, Cholesky, LDLT or QR-factorization methods, among others, depending on the characteristics of matrix **A** [35]), it may be more efficient to implement other algorithms.

The numerical stability of the solution in linear algebra may be evaluated by the condition number and the numerical rank of matrix **A**. The condition number is defined as: ${k}_2\left(\mathbf{A}\right)=\frac{\sigma_{max}}{\sigma_{min}}$; a low condition number usually means that the system is well-conditioned. The rank (r) of a matrix is the number of linear independent rows (or columns) (r ≤ min {m, n}) and is equal to the number of singular values (*σ*_*i*_) in the matrix other than zero. When r = min {m, n} the matrix has full range, otherwise it is rank-deficient. If **A** is nearly rank-deficient (*σ*_*min*_ is small), then the solution **x** is ill-conditioned and possibly very large. A more robust solution to obtain the effective rank may be to evaluate the number of singular values of **AA**^**H**^ or **A**^**H**^**A** above a specified tolerance. Analysing the condition number and the rank of a matrix may make it possible to select the best method for system solution.

### Least squares

In general, although the normal equation is the fastest method it is not used to solve systems by LS as it yields worse numerical results than other methods. In the normal equation, accuracy depends on ${k}_2\left({\mathbf{AA}}^{\mathbf{H}}\right)={k}_2^2\left(\mathbf{A}\right)$, although this method may be used if **A** is well-conditioned [36]. If **A** is rank-deficient, then **x** = **A**\**B** is not necessarily the minimum norm solution. The more computationally expensive ***x*** *= pinv(****A***)*B computes the minimum norm least-squares solution. Specifically, the function *pinv(****A,*** *tol)* returns the Moore–Penrose pseudoinverse, obtained by SVD decomposition where the values above tolerance (tol) are set to zero; this may be adapted to an ill-conditioned problem (**A** is not of full rank). Another option to obtain the Moore–Penrose pseudoinverse is proposed in [37], which makes use of QR-factorization and an algorithm based on a reverse order law for generalized inverse matrices; this method was later refined in [38]. An iterative solution to obtain the Moore–Penrose pseudoinverse was published in [39].

### TLS

The TLS method implemented is the one that yielded the worst computational performance. This method performs SVD of the augmented matrix **C** ≔ [**A** : **b**]; If **C** is rank-deficient or nearly rank-deficient (its singular values decay gradually), it may be advisable to truncate its small singular values [40]. [41] presents basic information, references and applications for the TLS method. In [42], an interactive method is proposed which combines orthogonal projections to a sequence of generalized Krylov subspaces of increasing dimensions and Newton’s method. The introduction to [43] presents various alternatives to obtaining the solution using the TLS method and the authors present a solution based on randomized algorithms.

### Roots

Numerical solution of a polynomial is a classic problem in mathematical research [44]. Methods available with which to obtain the roots of a polynomial include Laguerre [45], Bairstow, Græffe and Müller, Horner, Jenkins and Traub, and Newton [46], etc., with differing performances in terms of accuracy, convergence and speed. The code presented uses the *roots()* function used by the QR-algorithm on the balanced Frobenius companion matrix to compute its eigenvalues.

### Eigenvalues and SVD

The eigenvalues of a square matrix **A** are the roots of its characteristic polynomial det(**A** − λ**I**) = 0. However, singular values of **A** are non-negative square roots of eigenvalues of (**A**^T^**A**), meaning that both methods are related. The general idea is to diagonalize the target matrix as the values of the diagonal are the eigenvalues. All methods to solve the eigenvalue problem are of an iterative nature [47]. The built-in MATLAB function eig(**A**) uses the generalized Schur decomposition method (implemented via the QR-algorithm or its variants), which consists of interactively obtaining an upper triangular matrix **U**, in which the values of its diagonal are the eigenvalues of **A**. The QR-algorithm can be adapted to small or moderately large non-symmetrical eigenvalue problems [48]. For large matrices, [49] provides possible alternatives.

### Prony-like methods

Other modifications have been made to the Prony method, generally with the intention of improving its numerical stability. If any of the parameters of equation (1) are known, the Prony method makes it easier to find a robust solution. In the polynomial method, small variations in the coefficients of equation (2) due to signal noise can result in large variations in its zeros and, consequently, the frequencies of the approximation will vary greatly. Parametric spectral estimation techniques, such as MUSIC (MUltiple SIgnal Classification) [50], ESPRIT (Estimation of Signal Parameters via Rotational Invariance Techniques) [51] or fast ESPRIT [52] offer an alternative that in many cases make it possible to obtain more robust solutions. [53] presents an algorithm for the factorization of a matrix pencil based on QR decomposition of a rectangular Hankel matrix, which simplifies the ESPRIT method.

The NAFASS (Non-orthogonal Amplitude Frequency Analysis of the Smoothed Signals) approach [54] makes it possible to obtain the set of frequencies that make up strongly correlated random sequences with *N* < 1500. [55] presents the physical interpretation of the Prony decomposition as a linear recording of memory effects that can exist in random sequences in which the Fourier decomposition is a partial case. [56] improves the NAFASS method, presenting a linear recurrence expression that obtains the set of frequencies.

Another way to obtain more robust results is to act on the signals before obtaining their decomposition in a Prony series by using pre-filtering [57]. In the modified instantaneous Prony method [58] the input data used in an application of speech parameter extraction are those derived from the signal x[n] instead of adjacent samples.

Applying the Prony method to a time window that can be moved along the x[n] signal makes it possible to perform time–frequency analysis. One such example could be the short-time matrix pencil method (STMPS) successfully used to obtain antennae responses [59]. The Piecewise Prony Method [60] essentially consists of dividing the signal to be interpolated into windows of variable length and sampling rate.

## Conclusions

Decomposition of a signal using Prony’s method can be considered a generalization of the Fourier decomposition. Although the method has been known since 1795, its application in engineering has only increased since about the 1970s as computer use has grown. This paper has presented the theoretical bases and their piece-by-piece implementation in MATLAB. It has also shown some of their limitations and the benefit of improving the quality of the mfVEP signals. With the information provided, readers can begin practical implementation of the most common Prony methods, test the reliability of the results and, if applicable, research other methods more appropriate to their areas of research.

## Abbreviations

AUC: Area under the ROC curve
DFT: Discrete Fourier transform
LS: Least squares
mfVEP: Multifocal visual-evoked potential
MPM: Matrix pencil method
MS: Multiple sclerosis
RMS: Root mean square
ROC: Receiver-operating-characteristic
SNR: Signal-to-noise ratio
SVD: Singular value decomposition
TLS: Total least squares
